# PSG diagnostic features of OSAHS in Chinese children: a retrospective cohort study

**DOI:** 10.1016/j.bjorl.2026.101920

**Published:** 2026-11-02

**Authors:** Li Wang, Xiaoqin Zhou, Junhua Shu, Ying Cheng, Yi Gan, Jingjing Ding, Huiping Zhang, Guirong Wang, Ying Liu, Huimin Li, Jie Huang, Danhong Zhu, Hongkun Zhao, Huihui Pi, Niefan Wang, Fang Wang

**Affiliations:** Department of Pediatrics, Maternal and Child Health Hospital of Hubei Province, Tongji Medical College, Huazhong University of Science and Technology, Wuhan, China

**Keywords:** Polysomnography, Children, Obstructive sleep apnea hypopnea syndrome, Oxygen saturation

## Abstract

**Objective:**

To evaluate the clinical utility of PSG in diagnosing and characterizing PSG features of OSAHS in children.

**Methods:**

A retrospective study was implemented from January 2024 to June 2025.A total of 106 children with sleep-related symptoms underwent standardized overnight PSG. Core indicators ‒ Apnea-Hypopnea Index (AHI), minimum oxygen saturation, sleep efficiency, N3 sleep stage proportion (slow-wave sleep ratio), and arousal index ‒ were analyzed.

**Results:**

The OSAHS group had higher AHI, ODI, and arousal index but lower N3 proportion and minimum SpO_2_ (all p < 0.05). No significant age-group differences were observed (p > 0.05). The mild OSAHS group showed higher sleep efficiency and minimum SpO_2_, and fewer arousals than moderate/severe groups (p < 0.05). OSAHS detection rate was significantly higher in snoring versus non-snoring children (p < 0.05). Obese children had significantly higher AHI, ODI, and arousal index than non-obese children (p < 0.05).

**Conclusion:**

PSG objectively reflects OSAHS severity in children. Children with OSAHS commonly exhibit sleep architecture disruption and hypoxia, which are associated with daytime functional impairments. PSG results guide individualized treatment planning plans.

## Highlights

• This study confirms that children with OSAHS present with disrupted sleep architecture, reduced N3 sleep and nocturnal hypoxia; PSG can objectively evaluate disease severity.
• Stratified analysis reveals differences in sleep efficiency, minimum SpO_2_ and arousal index between mild and moderate-to-severe pediatric OSAHS. Snoring and obesity are relevant risk factors.
• PSG parameters support the clinical value of polysomnography and provide objective evidence to guide individualized treatment for children with OSAHS.

## Introduction

Childhood Obstructive Sleep Apnea-Hypopnea Syndrome (OSAHS) represents a significant and multifaceted public health challenge, characterized by recurrent episodes of partial or complete upper airway obstruction during sleep, leading to disrupted sleep architecture, intermittent hypoxia, and hypercapnia. The reported prevalence of pediatric Obstructive Sleep Apnea-Hypopnea Syndrome (OSAHS) exhibits significant variation depending on the diagnostic criteria employed, underscoring the necessity of standardized definitions to facilitate accurate epidemiological comparison. Using overnight Polysomnography (PSG) with an Apnea-Hypopnea Index (AHI) threshold of ≥1 event per hour as the gold standard, community-based epidemiological studies suggest a prevalence range of approximately 1.2%–5.7% in the general pediatric population.^1^ Nighttime symptoms include habitual snoring, pauses in breathing, and restless sleep, while daytime manifestations include cognitive dysfunction such as attention deficits and academic difficulties. Without timely intervention, it may lead to long-term complications such as growth retardation, cardiovascular, and metabolic dysfunction.^2^ It is important to note that the clinical picture of pediatric OSAHS often lacks specificity, making it easy to confuse with common respiratory infections or behavioral abnormalities, which increases the difficulty of early identification.

Recent years have witnessed significant progress in research on the pathogenesis of pediatric Obstructive Sleep Apnea-Hypopnea Syndrome (OSAHS). Genetic factors are significant determinants of susceptibility to OSAHS, with gene polymorphisms in neurotransmitter systems such as serotonin and Gamma-Aminobutyric Acid (GABA) implicated in the regulation of the respiratory center.^3^ Additionally, anatomical factors such as craniofacial abnormalities, reduced upper airway volume, and malocclusion have been established as significant risk factors for OSAHS.^4^ Metabolic disorders, particularly insulin resistance and adipokine imbalance, also contribute to the pathophysiology of OSAHS. These studies suggest that pediatric OSAHS is not merely a simple biomechanical sleep disorder but rather a complex disease characterized by multisystem interactions.

Polysomnography (PSG) is recognized as the most reliable assessment method.^5^ By recording key parameters such as the Apnea-Hypopnea Index (AHI) and nocturnal saturation, it facilitates both definitive diagnosis and precise severity stratification. However, the clinical utility of this examination is constrained by several practical limitations, such as stringent equipment requirements, high operational costs, and limited patient tolerance, particularly among pediatric populations, which hinder its widespread adoption in primary healthcare units.^6^

Several critical knowledge gaps persist regarding the application of PSG in the diagnosis of pediatric OSAHS: First, reference ranges of PSG parameters for children of different age groups lack standardization; second, the quantitative relationship between PSG parameters and disease severity, obesity status, and snoring symptoms still needs further clarification; finally, the translation of PSG findings into individualized treatment plans remains a clinical issue that requires urgent attention.

Addressing these gaps, this study aims to systematically evaluate the clinical utility of PSG in the diagnosis of pediatric OSAHS, and characterize the specific PSG diagnostic features of OSAHS in children, providing evidence-based support for optimizing the treatment strategy for clinical management.

## Methods

### General information

This retrospective observational study was conducted and reported in accordance with the Strengthening the Reporting of Observational Studies in Epidemiology (STROBE) guidelines. The study period spanned from January 1, 2024, to June 30, 2025, and the data were extracted from the clinical database of the institution's Sleep Medicine Center. This study was approved by the Medical Ethics Committee of the institution (Ethics Approval Number: 2025-269-01). Due to the retrospective nature of the study, informed consent from the participants (children) and their legal guardians was waived.

### Retrospective cohort identificationand inclusion/exclusion criteria

Inclusion criteria: (1) Preschool and school-aged children aged 3–12 years; (2) Some children exhibited snoring symptoms for ≥ 3-months, accompanied by at least one of the following observable signs: daytime inattention or hyperactivity; abnormal sleeping positions; (3) Seeking medical attention due to rhinitis, adenoid hypertrophy, or obesity.

Exclusion criteria: (1) Presence of severe underlying diseases such as congenital heart disease or cerebral palsy; (2) Recent upper respiratory tract infection (within 1-month); (3) Long-term use of glucocorticoids or sedative medications; (4) Presence of contraindications for sleep monitoring, such as skin allergies that cannot be tolerated, severe anxiety or fear; (5) Insufficient monitoring data.

### Diagnostic criteria and group definitions

OSAHS diagnostic criteria: Based on the 2012 American Academy of Sleep Medicine (AASM) pediatric OSAHS diagnostic criteria, AHI ≥ 1 event/h, accompanied by at least one of the following: (1) A decrease in blood oxygen saturation ≥3%; (2) Sleep arousal; (3) Paradoxical thoracoabdominal breathing movements. According to the above criteria, 106 children were divided into the OSAHS group (n = 98) and the non-OSAHS group (n = 8).

OSAHS severity grading: Severity was graded based on AHI values as follows: mild (1 ≤ AHI <5 events/h), moderate (5 ≤ AHI < 10 events/h), and severe (AHI ≥ 10 events/h).^7^

Age grouping: Preschool (3–6 years) and early school age (7–12 years).

Obesity definition: Defined according to the Chinese pediatric obesity diagnostic criteria, a Body Mass Index (BMI) ≥ the 95th percentile for children of the same age and sex is defined as obesity.^8^

Snoring definition: Based on guardian reports, loud snoring occurring ≥3 nights per week is defined as habitual snoring (see Fig. 1 for the flowchart of patient selection).

**Fig. 1:**
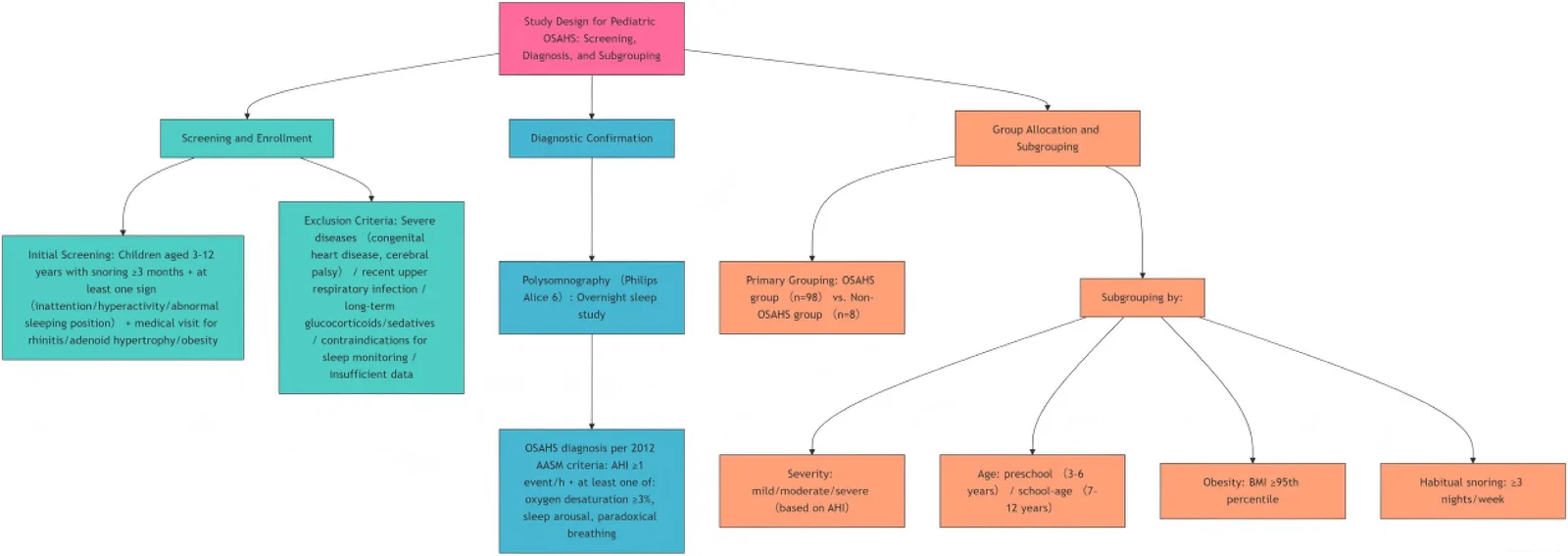
Illustrates the flowchart of patient selection.

The Philips Alice 6 polysomnography system was used for overnight sleep assessment. Certified sleep technicians provided standardized guidance to the children and their parents before monitoring. Participants were instructed to remain awake on the day of monitoring, avoid consuming caffeinated beverages and central nervous system medications 24-hs prior to the examination, and limit vigorous exercise before bedtime. The equipment was strictly calibrated before monitoring, confirming the normal functioning of all leads and sensors, and electrodes were placed according to standard procedures, which included 6-lead electroencephalogram (covering frontal, central, and occipital areas), a dual-lead electrooculogram, a chin electromyogram, a single-lead electrocardiogram, and an anterior tibialis electromyogram, along with a nasal airflow sensor, chest-abdominal respiratory movement monitoring belt, snoring detector, and pulse oximeter probe. All monitoring was conducted in a professional sleep laboratory that meets international standards, with environmental parameters controlled at a temperature of 22–24 °C, humidity of 50%–60%, and sound-light isolation conditions, recordings continued for no less than 7-hs to collect various physiological parameters, obstructive respiratory event index, minimum blood oxygen saturation, heart rate variability, and snoring characteristics.

PSG data were analyzed and processed following an internationally standardized protocol: first, automated initial screening was conducted via professional sleep analysis software, followed by a double-blind review performed by two physicians, each possessing more than five years of experience in pediatric sleep medicine, in accordance with the latest version (v2.6) of the AASM pediatric sleep event interpretation standards. In instances of discrepancy, a third senior physician rendered the final determination. To ensure the reliability of the results, all analysts regularly participated in annual standardized training.

### Observation indicators

1) Comparison of core indicators between children with OSAHS and those without OSAHS, including the Apnea-Hypopnea Index (AHI): the number of apnea and hypopnea events per hour of sleep; the lowest peripheral oxygen saturation (lowest SpO_2_): the minimum oxygen saturation value recorded during sleep; the Oxygen Desaturation Index (ODI): the frequency per hour of sleep that oxygen saturation drops by ≥3%; the proportion of N3 sleep stage (slow-wave sleep proportion): the percentage of the total time spent in N3 sleep stage of the Total Sleep Time (TST), N3 = Stage N3 sleep; the arousal index: the number of arousals per hour of sleep; sleep efficiency: the percentage of total sleep time relative to time in bed.
2) 106 children were stratified by age into ‒ preschool (3–6 years) with 28 cases, including 26 cases with OSAHS, early school age (7–9 years) with 78 cases, including 72 cases with OSAHS, comparing PSG monitoring parameters: AHI, longest apnea duration, REM proportion, and obesity comorbidity rate.
3) 98 children diagnosed by PSG were classified according to severity of condition based on AHI ‒ mild (42 cases), moderate (32 cases), severe (24 cases), comparing changes in monitoring parameters. Mild: 1 ≤ AHI < 5 times/h; moderate: 5 ≤ AHI < 10 times/h; severe: AHI ≥ 10 times/h.
4) Analyze the occurrence of OSAHS in snoring and non-snoring children, and the status of various monitoring parameters.
5) Analyze PSG parameters between obese and non-obese pediatric patients with OSAHS.

### Statistical processing

Data were organized and calculated using SPSS 24.0 statistical software. For continuous variables that conformed to a normal distribution, data are presented as mean ± Standard Deviation (SD), and independent samples *t*-tests were used for comparisons between two groups, while one-way analysis of variance (ANOVA) was employed for comparisons among three or more groups. When the assumption of homogeneity of variance was satisfied, post-hoc tests were performed using the Least Significant Difference (LSD) method; if homogeneity of variance was not met, Welch's ANOVA and Games-Howell post-hoc tests were used. Categorical variables were expressed as frequency and proportion [n (%)], and comparisons between groups were performed using Pearson's Chi-Square test or Fisher's exact test (when the expected frequency was <5). All hypothesis tests were two-sided, with a p-value < 0.05 considered statistically significant.

## Results

### Parameter comparison between OSAHS and non-OSAHS groups

The AHI, ODI, and arousal index of children in the OSAHS group were higher than those in the non-OSAHS group, while the proportion of N3 phase and the lowest SpO₂ were relatively lower (p < 0.05). See Table 1 for details.

**Table 1:** Comparison of parameters between OSAHS and non-OSAHS (x ± s).

Group	n	AHI (times/h)	Lowest SpO_2_ (%)	ODI (times/h)	Arousal index (times/h)	Proportion of N3 phase (%)
OSAHS	98	8.75 ± 2.39	84.62 ± 7.95	5.21 ± 1.89	18.34 ± 4.68	14.72 ± 6.57
Non-OSAHSS	8	0.53 ± 0.14	95.84 ± 8.27	0.35 ± 0.11	7.52 ± 1.51	23.15 ± 5.23
*t*		9.683	3.826	7.240	6.486	3.532
p		<0.001	<0.001	<0.001	<0.001	0.001

Note: OSAHS, Obstructive Sleep Apnea-Hypopnea Syndrome; AHI, Apnea-Hypopnea Index; SpO_2_, Peripheral Oxygen Saturation; ODI, Oxygen Desaturation Index; N3, Stage N3 sleep.

### Comparison of parameters in preschool and early school-age children

There are small differences in the parameters among children of different age groups (p > 0.05). See Table 2 for details.

**Table 2:** Comparison of parameters in preschool and early school-age children with OSAHS (x ± s).

Group	n	AHI (times/h)	Lowest SpO_2_ (%)	Arousal index (times/h)	Proportion of N3 phase (%)
Preschool	26	5.42 ± 1.82	84.76 ± 4.11	16.25 ± 3.12	16.85 ± 4.21
Early school-age	72	4.98 ± 1.15	85.36 ± 3.47	14.87 ± 4.74	17.29 ± 5.11
*t*		0.671	0.401	1.152	0.280
p		0.504	0.689	0.252	0.780

Note: OSAHS, Obstructive Sleep Apnea-Hypopnea Syndrome; AHI, Apnea-Hypopnea Index; SpO_2_, Peripheral Oxygen Saturation; N3, Stage N3 sleep.

### Comparison of parameters at different severity levels

The sleep efficiency and minimum SpO_2_ of children with mild OSAHS are higher than those of children in the moderate and severe groups, and the number of awakenings is lower than that of the other two groups (p < 0.05). See Table 3 for details.

**Table 3:** Comparison of parameters at different severity levels (x ± s).

Group	n	AHI (times/h)	Minimum SpO_2_ (%)	Sleep Efficiency (%)	Arousal Index (times/h)
Mild	42	2.82 ± 0.63	89.19 ± 3.82	85.26 ± 6.86	12.16 ± 3.34
Moderate	32	6.92 ± 1.32	82.75 ± 4.91	80.73 ± 7.97	19.64 ± 5.25
Severe	24	18.45 ± 3.75	72.36 ± 8.57	76.45 ± 9.24	26.82 ± 8.91
F		15.362	42.514	45.228	21.254
p		<0.001	<0.001	<0.001	<0.001

Note: Post-hoc tests (LSD method) revealed statistically significant differences in all parameters between the mild group and both the moderate and severe groups (p < 0.05); statistically significant differences in AHI, minimum SpO, and arousal index were also observed between the moderate and severe groups (p < 0.05).

SpO_2_, Peripheral Oxygen Saturation.

### Comparison of OSAHS occurrence and monitoring parameters between snoring and non-snoring children

The detection rate of OSAHS in snoring children is significantly higher than that in non-snoring children (p < 0.05). See Table 4 for details.

**Table 4:** Comparison of OSAHS occurrence and monitoring parameters between snoring and non-snoring children.

Group	n	OSAHS detection rate	AHI (times/h)	Lowest SpO₂ (%)
Snoring	89	85 (87.64)	6.82 ± 1.25	86.34 ± 7.55
Non-snoring	17	13 (76.47)	1.25 ± 0.32	93.87 ± 4.16
*t*/χ^2^		7.412	12.518	2.778
p		0.006	23.53	0.007

Note: OSAHS, Obstructive Sleep Apnea-Hypopnea Syndrome; AHI, Apnea-Hypopnea Index; SpO_2_, Peripheral Oxygen Saturation.

### Comparison of PSG parameters between obese and non-obese pediatric patients

The AHI, ODI, and arousal index of obese children’s patients are significantly higher than those of non-obese children’s patients (p < 0.05). See Table 5 for details.

**Table 5:** Comparison of PSG parameters between obese and non-obese pediatric patients with OSAHS (x ± s).

Group	n	AHI (times/h)	ODI (times/h)	Arousal Index (times/h)	Proportion of N3 phase (%)
Obese	39	10.32 ± 2.52	8.66 ± 2.15	22.45 ± 6.29	12.34 ± 3.71
Non-obese	59	5.24 ± 1.82	3.79 ± 1.22	14.85 ± 4.68	17.25 ± 4.41
*t*		12.046	14.859	7.123	5.961
p		<0.001	<0.001	<0.001	<0.001

Note: PSG, Polysomnography; OSAHS, Obstructive Sleep Apnea-Hypopnea Syndrome; AHI, Apnea-Hypopnea Index; ODI, Oxygen Desaturation Index; N3, Stage N3 sleep.

## Discussion

Pediatric OSAHS is a sleep-disordered breathing condition that is increasingly recognized in pediatric clinical practice, differing fundamentally in pathogenic mechanisms and pathophysiological characteristics from those observed in adult patients. As the medical community gains a deeper understanding of the impact of sleep quality on children, the early and accurate diagnosis of OSAHS has become an important topic in current clinical research.^9^ In routine diagnosis and treatment, physicians often base their judgments on the clinical picture and routine health examination findings of the child; however, due to the unique anatomical characteristics of children's respiratory tracts, their symptoms often manifest atypically, making subjective assessment prone to diagnostic bias.^10^

The results of this study show that the AHI, ODI, and arousal index in children in the OSAHS group were significantly higher than those in the non-OSAHS group, while the percentage of time spent in N3 sleep and the lowest SpO_2_ were significantly lower. Notably, the arousal index in the OSAHS group (18.34 ± 4.68 times/h) was significantly higher than that in the non-OSAHS group (7.52 ± 1.51 times/h), suggesting that pediatric OSAHS patients exhibit significant sleep fragmentation, which may be related to compensatory responses of the central nervous system to respiratory events in children, differing from the hypoxia-dominated pathological pattern observed in adult patients.^10^ Regarding the influence of age on PSG parameters, this study found no statistically significant differences in the measured parameters between preschool and early school-age children (p > 0.05). Potential explanations for these findings may include limitations in sample size, variations in inclusion criteria, and individual differences in the degree of lymphoid tissue hyperplasia among children of different age groups.^10^ Future longitudinal studies with larger sample sizes are needed to further clarify the impact of age factors on PSG parameters.

As the gold standard diagnostic modality in the field of sleep medicine, Polysomnography (PSG) is governed by well-established application standards in the assessment of adult sleep disordered breathing; however, its application in the pediatric population is complicated by dynamic growth and developmental changes, presenting several challenges warranting in-depth investigation.^7^ Current challenges in the clinical implementation of PSG for diagnosing pediatric OSAHS include the adaptability of monitoring equipment to the special physiological characteristics of children, compliance management for younger patients, and the establishment of specific diagnostic thresholds for different age groups, all of which hinder improvements in diagnostic accuracy and the formulation of optimal treatment strategies.

Children with OSAHS exhibit multidimensional pathophysiological characteristics. PSG parameter analysis shows that the AHI, ODI, and arousal index of children in the OSAHS group are higher than those in the non-OSAHS group, and the proportion of N3 sleep and the lowest SpO_2_ are significantly lower (p < 0.05). Specifically, children in the OSAHS group presented typical features ‒ significantly increased AHI, ODI, and arousal index, while the proportion of N3 sleep and the lowest SpO_2_ are significantly decreased. This result is consistent with the concept of “triad of pediatric sleep-disordered breathing” proposed by the international sleep medicine community in recent years, which indicates that ventilation disorders, sleep fragmentation, and hypoxia interact with each other, forming a vicious cycle. It should be noted that the synchronous increase in the arousal index and AHI may reflect the compensatory response of the central nerve system in children to respiratory events, which is significantly different from the hypoxia-dominated pathological pattern in adult patients. Studies indicate that differences in these parameters across different pediatric age groups were not statistically significant (p > 0.05).

Stratified analysis of monitoring data by disease severity revealed that children in the mild group had significantly higher sleep efficiency and lowest SpO_2_, as well as fewer awakenings, compared to those in the moderate and severe groups (p < 0.05). These characteristics provide a theoretical basis for formulating age-specific intervention strategies. In contrast, during the school-age period, children often exhibit disordered sleep architecture.^8^ These findings suggest that as age increases, the focus of OSAHS may shift from ventilatory function to neural regulation. However, in previous studies, preschool children often exhibit unique characteristics of high AHI and relatively high SpO_2_, which are related to the anatomical characteristics of active lymphoid tissue hyperplasia in this age group.

Analysis indicates that the physiological sleep characteristics of children with OSAHS of different severity show progressive changes. Children with mild OSAHS maintain relative stability in sleep maintenance ability and oxygenation status, whereas progression to moderate and severe stages is associated with synergistic deterioration of multiple physiological parameters.^11^ Notably, the degree of decline in sleep efficiency exhibits a threshold relationship with the arousal index ‒ when the AHI exceeds the critical value of 10 events/h, sleep quality shows a precipitous decline. A critical threshold exists in the pathophysiology of pediatric sleep-disordered breathing; once this threshold is exceeded, this triggers compensatory dysregulation of sleep architecture.^12^^,^^13^

In examining the association between clinical features and OSAHS, this study found that the prevalence of OSAHS in children with snoring (95.51%) was significantly higher than that in non-snoring children (76.47%), but 4.49% of snoring children still did not meet the diagnostic criteria for OSAHS. This suggests that although snoring is an important clinical manifestation of OSAHS, its predictive value is limited, and relying solely on snoring symptoms may lead to underdiagnosis or misdiagnosis. In addition, the AHI, ODI, and arousal index were significantly higher in obese children compared to non-obese children, while the proportion of N3 sleep was significantly lower, indicating that obesity has a multifactorial impact on the severity of OSAHS. This may be related to visceral adipose tissue secreting inflammatory mediators such as Interleukin-6 (IL-6) and Tumor Necrosis Factor-alpha (TNF-α), which in turn alter the sensitivity of respiratory center regulation.^14^, ^15^, ^16^ Studies indicated that dopamine levels and genetic variations in DRD2 rs1800497, HTR2A rs2770304 polymorphism, as potential modulators of its occurrence and severity or co-occurring sleep conditions.^17^^,^^18^ Compared to previous studies, the innovations of this study are as follows: first, a systematic analysis of the quantitative relationships between different clinical features (age, severity, obesity status, snoring condition) and Polysomnography (PSG) parameters; second, clarification of the characteristics of sleep architecture disruption in children with OSAHS, particularly the synchronous increase in arousal index and AHI; third, validation of the threshold effect between PSG parameters and disease severity, providing quantitative evidence for clinical decision-making.

PSG offers multidimensional advantages in application in the clinical management of childhood OSAHS. PSG can accurately quantify the frequency of respiratory events, fluctuations in blood oxygen saturation, and sleep structure parameters, which not only allows for objective grading of ventilatory dysfunction but also effectively distinguishes between obstructive and central apnea events, addressing the limitations of conventional examination methods.^19^^,^^20^ The multi-parameter analysis provided by PSG offers a reliable basis for clinicians to develop individualized intervention plans, such as more accurately determining the indications for adenoidectomy, predicting the effects of continuous positive airway pressure treatment, and optimizing respiratory support parameter settings.^21^^,^^22^

This study has the following limitations: First, this study is a single-center retrospective study with a single source of samples, which may have selection bias, and the generalizability of the research findings is somewhat limited. Second, a marked disparity in sample sizes exists between the OSAHS group (98 cases) and the non-OSAHS group (8 cases), which may affect the statistical power of the comparison between the two groups. Third, this study did not conduct long-term follow-up of the children, thereby precluding the assessment of the relationship between Polysomnography (PSG) parameters and long-term prognosis. Fourth, information such as snoring and daytime symptoms was obtained via guardian reports, introducing the possibility of recall bias. Fifth, this study did not perform peripheral carbon dioxide testing, and some cases with hypercapnia as the primary clinical presentation were not included in the case group; Sixth, this study did not include a healthy control group, so it is unable to establish a normal reference value range for pediatric PSG parameters. Future research should be designed as multi-center, prospective cohort studies, include larger sample sizes, incorporate extended longitudinal follow-up to further validate the conclusions of this study, and establish reference standards for PSG parameters based on the Chinese pediatric population.

## Conclusion

This study confirms that children with OSAHS have significantly more adverse PSG parameters than those without, including higher AHI, prolonged oxygen desaturation events, and more disrupted sleep. Obesity is identified as a key risk factor, with children with OSAHS and comorbid obesity showing more severe abnormalities. Habitual snoring is a strong predictor of OSAHS. PSG parameters effectively differentiated different severity levels of OSAHS. Furthermore, the disease profile differed between preschool and school-aged children, suggesting age-specific etiologies. These results support the use of PSG for evaluation and the need for tailored management in high-risk pediatric groups.

## Authors’ contributions

Li Wang and Fang Wang: Conceptualization; Methodology; Investigation; Data curation, formal analysis; Writing-original draft preparation; Writing-reviewing and editing. Li Wang, Xiaoqin Zhou, Junhua Shu, Ying Cheng, Yi Gan, Jingjing Ding, Huiping Zhang, Guirong Wang, Ying liu, Huimin Li, Jie Huang, Danhong Zhu, Hongkun Zhao, Huihui Pi and Niefan Wang: Methodology; Investigation; Data curation. Li Wang, Xiaoqin Zhou, Junhua Shu, Yi Gan: Conceptualization; Investigation; supervision, resources. Li Wang: Conceptualization, supervision, resources; Writing-original draft preparation, Writing-reviewing and editing. Fang Wang: Conceptualization, supervision, funding acquisition, resources; Writing-original draft preparation; Writing-reviewing. Ying Cheng: Funding acquisition, resources. All authors approving the final version of the manuscript.

## Consent for publication

Not applicable.

## Ethics approval and consent to participate

This study was conducted in accordance with the declaration of Helsinki. This study was conducted with approval from the Ethic committee of Maternal and Child Health Hospital of Hubei Province, Tongji Medical College, Huazhong University of Science and Technology (protocol number: 2025-269-01, approval date: December 19, 2025).

## Declaration of Generative AI and AI-assisted technologies in the writing process

During the preparation of this work the authors used Helixlife (helixlife.cn) in order to improve readability and language. After using this tool/service, the authors reviewed and edited the content as needed and take full responsibility for the content of the published article.

## Funding

The study was supported by China Foundation for International Medical Exchanges (Z-2019-41-2201). None of the funding body had any influence on study design, implementation, data analysis, or interpretation.

## Data availability statement

The datasets used and/or analyzed during the current study available from the corresponding author on reasonable request.

## Declaration of competing interest

The authors declare no conflicts of interest.
